# Toll-Like Receptors and Cytokines as Surrogate Biomarkers for Evaluating Vaginal Immune Response following Microbicide Administration

**DOI:** 10.1155/2008/534532

**Published:** 2008-12-22

**Authors:** Sadhana M. Gupta, Clara C. Aranha, Madhu C. Mohanty, K. V. R. Reddy

**Affiliations:** Immunology Division, National Institute for Research in Reproductive Health, J.M. Street, Parel, Mumbai 400012, India

## Abstract

Topical microbicides are intended for frequent use by women in reproductive age. Hence, it is essential to evaluate their impact on mucosal immune function in the vagina. In the present study, we evaluated nisin, a naturally occurring antimicrobial peptide (AMP), for its efficacy as an intravaginal microbicide. Its effect on the vaginal immune function was determined by localizing Toll-like receptors (TLRs-3, 9) and cytokines (IL-4, 6 , 10 and TNF-*α*) in the rabbit cervicovaginal epithelium following intravaginal administration of high dose of nisin gel for 14 consecutive days. The results revealed no alteration in the expression of TLRs and cytokines at both protein and mRNA levels. However, in SDS gel-treated group, the levels were significantly upregulated with the induction of NF-*κ*B signalling cascade. Thus, TLRs and cytokines appear as sensitive indicators for screening immunotoxic potential of candidate microbicides.

## 1. INTRODUCTION

Topical microbicides are being sought for preventing the transmission of human immunodeficiency virus (HIV) infection and other sexually transmitted infections (STIs) [1]. The success of anti-HIV/STI microbicides depends on their ability to maintain natural vaginal defenses, since the stratified epithelium of human vagina in unison with the microflora provides an efficient barrier against HIV-1 [2]. It is known that inflammatory conditions induced by pathogens or chemical irritants may lead to a higher risk of acquiring and transmitting HIV-1 due to epithelial disruption and recruitment of HIV-1 by immune cells [3].

During early efforts to develop safe and effective topical microbicides to eliminate the risk of sexual transmission of STIs/HIV-1, Nonoxynol-9 (N-9), a chemical-based product, emerged as the lead candidate due to its potent activity against microbes *in vitro* and widespread commercial use as a contraceptive [4, 5]. However, clinical trials revealed that frequent use of N-9 resulted in inflammatory lesions and ulceration of the genital tract mucosa creating a direct portal of entry for HIV-1 [6, 7]. Two other products, cellulose sulfate (6%) and carraguard also failed as microbicides despite preclinical safety trials (http://www.aidsmap.com).

One of the most promising alternatives to chemical-/detergent-based vaginal microbicides appears to be the naturally occurring antimicrobial peptides (AMPs) [8, 9]. They represent ancient host defense effector molecules produced by the innate immune system in response to pathogenic microbes or inflammatory stimuli. The most extensively characterized AMP is the lantibiotic nisin, a 34 amino acid cationic, amphiphilic peptide having a molecular mass of 3.5 kDa [10, 11]. Our earlier studies have shown that intravaginal administration of nisin does not trigger any inflammatory response in CVE of rabbits [8, 12].

In the context of developing a safe microbicide, it is also essential to determine its interference with the vaginal mucosal immune function. A remarkable feature of the immune cells of the vaginal mucosa is their ability to recognize and discriminate invading microbes from commensal bacteria or inflammatory agents [13, 14]. The vaginal mucosal immune cells are equipped with different Toll-like receptors (TLRs), which belong to a large family of highly conserved proteins that are essential pathogen-specific recognition sensors of the innate immune system [15–18]. So far, 11 members of this family have been identified on various cells [19–21]. TLR signalling is a key component of communication between vaginal epithelial cells and underlying immune cells in the lamina propria [20, 22, 23]. Therefore, we presumed that TLRs may act as suitable biomarkers for assessing vaginal inflammation induced by candidate microbicides.

A critical gap in microbicide development has been caused due to the absence of surrogate safety markers. Since vaginal microbicides would be used repeatedly over decades, it would be advantageous to evaluate their effects on vaginal innate immune defense system. Suitable biomarkers are required to determine the changes that take place in the mucosal environment at molecular level, including induction of inflammation or loss of host vaginal defenses, which are not reflected by histology [23]. A noninvasive method to detect subtle tissue inflammation would be a helpful adjunct to the histological findings. Considering the pivotal role of TLRs and cytokines in vaginal mucosal innate immunity, we carried out a study to determine the vaginal immune response after intravaginal application of nisin gel.

For this study, we selected TLRs-3 and 9, and cytokines (IL-4, 6, 10, and TNF-*α*) as they play an indispensable role in host vaginal innate and adaptive immunity. TLRs-3 and 9 are known to be expressed on plasma membrane as well as within the endosomes and stimulate memory B-cell development, proliferation, and differentiation. They also induce the secretion of anti-inflammatory cytokines from mucosal epithelium when they sense inflammatory stimuli [19]. The objectives of the present study were to (1) examine the effect of repetitive application of high dose of nisin gel on vaginal mucosal immunity using rabbit model; (2) investigate changes induced by nisin gel in the TLR and cytokine milieu of rabbit vagina; and (3) determine the role of TLRs and their downstream signalling cascade in host vaginal defense mechanism. To the best of our knowledge, this is the first report to demonstrate the *in vivo* distribution of TLRs and identify putative pathways driven by TLR cytokine interactions in the CVE of rabbit after intravaginal administration of microbicide, nisin gel.

## 2. MATERIALS AND METHODS

Nisin (Sigma Aldrich, St. Louis, USA, Lot *#* 075K1355) was purified by gel filtration chromatography on Sephadex G-25 and RP-HPLC as described [24]. Briefly, after initial purification by gel filtration chromatography, the lyophilized sample was acidified with 0.1% trifluoroacetic acid (Merck Ltd, Mumbai, India) and applied onto a preparative C8 column (Vydac, Ill, USA) connected to a Dionex HPLC unit. Peptide fractions were eluted in a three-step gradient of 0.01% trifluoroacetic acid in water and 0.01% trifluoroacetic acid in 50% acetonitrile (Spectrochem, Mumbai, India): 0–100% (60 minutes), held at 100% for 5 minutes and brought back to 0% (100-0%). The individual peptide peaks were collected and their absorbance was monitored at 220 nm using spectrophotometer (Shimadzu, UV-160, and Japan). The positive fractions were pooled and concentrated by lyophilization. Nisin gel (15.15 mM equivalent to 50 mg) was formulated using 1% polycarbophil as a gelling agent, SDS gel (56 *μ*M equivalent to 32 *μ*g) in 1% polycarbophil, and placebo gel using 1% polycarbophil.

### 2.1. Animals

Sexually mature female Belgium White Rabbits (mean age 7 ± 1 months; mean body weight, 2.70 ± 0.40 kg) were maintained under standard laboratory conditions (temperature 20 ± 1°C, relative humidity 50 ± 10%, and 12 hours light:12 hours darkness cycle). Animals were housed individually in stainless steel cages; food and water were made available *ad libitum*. The study was approved by the Ethics Committee of National Institute for Research in Reproductive Health (NIRRH), Parel, and Mumbai. All measures taken were in accordance with approved guidelines of Committee for the purpose of Control and Supervision of Experiments on Animals (CPCSEA), established by Government of India on animal care.

### 2.2. Intravaginal administration of nisin gel in rabbits

Rabbits were divided into three groups each consisting of six animals. First group of animals received nisin gel formulation prepared in 1% polycarbophil (15.15 mM in 2 mL of 1% polycarbophil/day for 14 consecutive days). The second and third groups of rabbits received SDS gel (56 *μ*M in 2 mL of 1% polycarbophil/day/14 consecutive days) and 2 ml of 1% polycarbophil gel, respectively. The gel formulations were delivered into the vagina using a 12-cm flexible catheter, inserted up to its 8 cm mark. The body weights of rabbits and clinical observations were recorded daily, including swollen vulva areas, blood stained urine, and soft stools.

On day 15, the cervicovaginal lavages (CVLs) were collected from placebo and treated rabbits by administering 500 *μ*l of saline and animals were autopsied using CO_2_ asphyxiation. Samples were centrifuged at 1000 g for 5 minutes to separate supernatant from cell debris. The vaginal tissues were slit open ventrally between the urethral orifice and fornix. Portions of cervicovaginal tissues were fixed in Bouin's solution. Paraffin sections of 5 *μ*m thick were cut and utilized for immunolocalization of TLRs (TLR-3 and 9) and cytokines (IL-4, 6, 10, and TNF-*α*). The remaining tissues were processed immediately for flow cytometric evaluation of TLRs and RT-PCR analysis.

### 2.3. Isolation of rabbit CVE cells

CVE tissue was isolated from three groups of rabbits, cut into small fragments, and pooled (200–300 mg) groupwise prior to being snap frozen on dry ice and stored in liquid nitrogen until further analysis. On the day of analysis, cell suspensions were prepared by enzymatic digestion as described previously [25]. Briefly, fragments of CVE were digested with 50 000 U/mL of trypsin (Sigma Aldrich, St. Louis, USA) in 2.5% (w/v) pancreatin solution (Sigma) for 1 hour at 4°C, followed by 1 hour digestion at 22°C with constant rotation (120 rpm). Digested tissues were vortexed to release the epithelial sheets before passing cell suspension through a 20 *μ*m nylon mesh (Millipore Corp., Billerica, MA, USA). Cells were aspirated through 20 gauge needles to prepare isolated cells before resuspension in complete Rosewell Park Memorial Institute-1640 (RPMI-1640) medium (Life Technologies, Inc., Md, USA) (25 mM HEPES supplemented with 10% fetal bovine serum (Hyclone), 50 *μ*M 2-mercaptoethanol, 2 mM L-glutamine, 100 *μ*g/mL streptomycin, and 100 U/mL penicillin) and then seeded onto Nunc cell culture inserts (diameter, 10 mm, pore size, 0.4 *μ*m) coated with diluted Matrigel (Sigma) at 300 *μ*l/apical chamber of the cell culture insert. Inserts were placed into 24 well tissue culture plates containing 500 *μ*l of culture media per well. The purity of epithelial cell cultures was evaluated by using anti-CD45 antibody which showed that CVE cells accounted for more than 90% of the cells present on each insert.

### 2.4. Immunofluourescent (IF) localization of TLRs

The purpose of this experiment was to determine the pattern of changes in TLR expression in CVE of placebo, nisin, and SDS gel-treated rabbits. CVE tissue sections were deparafinized in xylene, rehydrated in descending grades of alcohol, and finally washed in PBS (0.1 M PBS, pH 7.4). The nonspecific binding was blocked with 1:100 dilution of normal goat serum and incubated at 37°C for 1 hour. After washing in PBS, slides were incubated overnight at 4°C with 1:100 dilution of goat antihuman TLR-3 and TLR-9 antibody (Imgenex) (cat *#* SC-8691 for TLR-3 and cat *#* IMG 305 for TLR-9) in PBS. The sections were washed in PBS, and incubated with FITC conjugated donkey antigoat secondary antibody (Sigma Aldrich, St. Louis, USA) at a dilution of 1:1000 at 37°C for 1 hour in dark. Immunofluorescence signals were captured using laser scanning confocal microscopy (LSCM) (Zeiss, 510 meta, Germany).

### 2.5. Flow cytometric analysis

Flow cytometry was performed on a FACS vantage flow cytometer (Becton Dickinson, USA) to evaluate the surface expression of TLR-3 and 9 in CVE cells. Based on initial optimization studies, the cells (1 × 10^6^ /mL) were washed briefly in PBS, and nonspecific binding was blocked with 1:100 dilution of normal goat serum at 37°C for 1 hour. For intracellular staining, cells were first treated with fixation buffer (4% pararformaldehyde) followed by permeabilization buffer (0.1% Triton X 100). After washing with PBS, slides were incubated overnight at 4°C with 1:100 dilution of goat antihuman antibody to TLR-3 and TLR-9 in 1% BSA in PBS. After washing in PBS, the cells were incubated at 37°C for 1 hour in dark with FITC conjugated donkey antigoat secondary antibody at a dilution of 1:1000. After washing five times in PBS, fluorescence signals for each cell were detected through a 520 nm argon-ion laser. We included normal goat serum as appropriate –ve control to rule out any nonspecific binding of antibody through Fc-receptors. Data for a minimum of 10 000 events was collected for each sample. Fluorescence histograms and dot plots were generated after gating the cell population using cell quest software (http://facs.scripps.edu/).

### 2.6. Immunohistochemical localization of cytokines

The immunohistochemical localization of proinflammatory cytokines (IL-6 and TNF-*α*) was carried out as per the kit protocol described by manufacturer (R&D systems, Minn, USA) (cat *#* DTA00C for IL-6 and cat *#* DY206 for TNF-*α*). The localization of IL-4 and 10 was performed using goat antihuman primary antibody (Sigma Aldrich, cat *#* I7526 for IL-4, I5020 for IL-10). Briefly, cervicovaginal tissue sections from placebo, nisin, and SDS gel-treated rabbits were processed as described above. The endogenous peroxidase activity was quenched by treating cervicovaginal tissues with 0.03% H_2_O_2_ in methanol for 10 minutes and rinsed in PBS for 5 minutes. To rule out nonspecific binding, sections were incubated in normal goat serum instead of primary antibody and considered as negative controls. After washing with PBS, slides were incubated with respective primary antibodies overnight at 4°C. Next day, slides were washed twice in PBS and incubated for 1 hour at RT with 1:100 dilution of donkey antigoat secondary antibody conjugated to horse radish peroxidase (HRP) (Sigma Aldrich, cat *#* G6638).

After washing in PBS, the sections were incubated for 10 minutes with 3-3′diaminobenzidine tetrahydrochloride (DAB) (Sigma Aldrich, St. Louis, USA) containing Ni^2+^ and H_2_O_2_ in PBS. The tissue sections were then briefly rinsed in water, counterstained with hematoxylin. After dehydration in alcohol series, sections were cleared in xylene, mounted with DPX, and visualized using Olympus BX50 microscope.

### 2.7. Image analysis

Semiquantitative analysis of the immunoprecipitates of cytokines was performed using image analysis software Biovis 1.42 as detailed previously [26]. Briefly, four areas from each section were randomly selected for each animal in the three groups. Images from these areas were grabbed by the CCD camera. The integrated optical density (IOD) of each area was calculated for the brown color immunoprecipitates. Negative control slides were analyzed in a similar fashion. The mean ± SDs were calculated for each group.

### 2.8. Measurement of cytokine levels in cervicovaginal lavages

Levels of proinflammatory (IL-6 and TNF-*α*), Th-2 type IL-4, and anti-inflammatory cytokine (IL-10) in CVLs were measured by ELISA as per the protocol discussed above. Optical densities of the product formed were measured by using ELISA reader (ELX 800, Bio-TEK Instruments). Cytokine levels were calculated by quadratic regression analysis based on logarithmically transformed optical densities. Interference of gel formulations in cytokine ELISA was ruled out by spiking known concentrations of standards. Control and treated samples were assayed in duplicate wells and the experiment was repeated thrice.

### 2.9. Measurement of Phospho-NF-*k*B p65 in CVE cells

The activation of NF-*k*B signalling cascade in CVE tissue was determined as per the manufacturer's protocol using Phospho-NF-*k*B p65 (Ser536) Sandwich ELISA kit (Cell Signaling, MA, USA). Briefly, cell lysates were prepared with lysis buffer (20 mM tris,150 mM NaCl, 1 mM EDTA,1 mM ethylene glycol-bis(2-aminoethyl)-N,N,N′,N′ (EGTA), 1% Triton X-100, 2.5 mM sodium pyrophosphate, 1 mM *β*-glycerophosphate, 1 mM Na3 Vo4, 1 *μ*g/mL leupeptin) and sonicated on ice. The lysates were centrifuged at 14 000 rpm (Eppendorf 541R) for 10 minutes at 4°C to pellet the cell debris. The supernatants were collected, aliquoted, and stored at −80°C in single use aliquots. Cell lysates were added to microwells coated with Phospho-NF-*k*B p65 (Ser536) (93H1) mouse mAb and incubated overnight at 4°C.

Following extensive washing with wash buffer, 100 *μ*l of Phospho-NF-*k*B p65 rabbit mAb (Cell Signaling, MA, USA, cat *#* 3033) was added to the wells and incubated for 1 hour at 37°C to detect the captured Phospho-NF-*k*B p65 protein. After washing, 100 *μ*l antirabbit IgG secondary antibody coupled to HRP (Cell Signaling, MA, USA, cat *#*7074) was added and incubated at 37°C for 1hour to recognize the bound antibody. 100 *μ*l of substrate, 3′-3′tetramethylbenzidine (TMB) was added and incubated for 10 minutes at 37°C to develop blue color. Reaction was stopped by adding 100 *μ*l of stop solution. The OD of yellow-colored product was measured at 450 nm by ELISA reader. Each value is the mean ± SD of six individual observations obtained from three experiments performed on different days.

### 2.10. RNA isolation and RT-PCR analysis of TLRs, cytokines, and defensin-1 molecules

Total RNA was isolated from CVE tissue from nisin, SDS, and placebo gel-treated rabbits by sequential extraction using TriPure reagent according to the manufacturer's protocol (Roche, Germany). RNA was quantitated spectrophotometrically at 260 and 280 nm (UV-160A, Shimadzu, Japan). The quality and integrity of RNA was determined by 1.5% denaturing formaldehyde agarose gel electrophoresis and used for semiquantitative RT-PCR. Prior to amplification, all RNA samples were treated with RNase-free DNase-I (Qiagen) to preclude genomic DNA contamination. Briefly, the reaction was carried out in a PTC-200 thermal cycler (MJ Research, Maryland, USA) in a total volume of 50 *μ*l reaction mixture containing 10 *μ*l 5X RT-PCR buffer, 2 *μ*l dNTP mixture, 1 *μ*l enzyme mix, 2 *μ*l of 0.4 *μ*M each primer, 1 *μ*l total RNA (2 *μ*g), 1 *μ*l DTT solution, and 31 *μ*l RNAse free water. RT PCR to synthesize cDNA was carried out at 50°C for 30 minutes followed by amplification using gene-specific primers (Table 1) for 30–40 cycles at 94°C for 30 seconds, annealing at 55–64°C for 30 seconds, extension at 68°C for 40 seconds followed by a final extension at 68°C for 7 minutes. Reaction was amplified in the absence of reverse transcriptase as negative control. A nontemplate control reaction was also included to ensure lack of DNA contamination. Housekeeping gene, GAPDH served as internal loading control. PCR products were electrophoresed through 1.5% agarose gel, stained with EtBr (0.5 *μ*g/mL) (Sigma Aldrich, St. Louis, USA), visualized by UV transillumination, and imaged using Gel Doc system (Bio-Rad, Calif, USA).

### 2.11. Statistical analysis

In most instances, the results were expressed as mean ± standard deviations. Statistically, significant differences between placebo and treatment groups were assessed using analysis of variance or *t* test, and differences were considered significant if *p* values were < .05.

## 3. RESULTS

### 3.1. Immunofluorescent localization of TLRs

**Figure 1:**
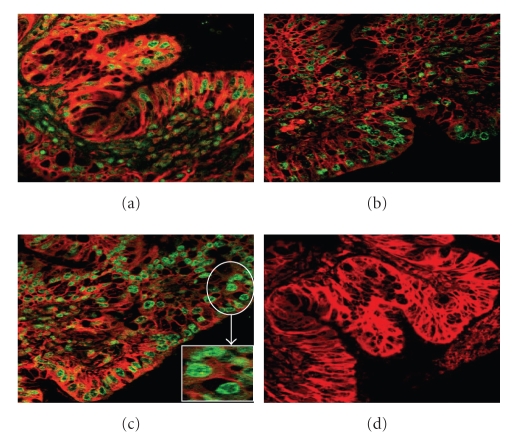
Laser Scanning Confocal Microscope (LCMS) images of TLR-9 protein expression in rabbit CVE after intravaginal administration of nisin (15.15 mM in 2 mL of 1% polycarbophil gel) (b), SDS (56 *μ*M in 2 mL of 1% polycarbophil gel), (c) and 2 mL of 1% placebo gel (a) for 14 consecutive days. Expression was found to be localized in the cytoplasm of cells. Note significant increase of TLR expression in SDS gel group (c) compared to placebo (a) and nisin gel administered (b) groups. Similar expression pattern was observed in TLR-5. The figures shown are the representative pictures from three independent experiments (Mag X 40; inset image X 630).

To determine the effect of nisin gel on mucosal immunity, rabbits were intravaginally administered with placebo, nisin, and SDS gels. Representative images of TLR-9 immunofluorescent localization of TLR-9 in CVE cells are shown in Figure 1. TLR-9 was localized on the cell membrane and also within the cytoplasm of CVE cells. Similar pattern of expression was seen for TLR-3 (data not shown). Vaginal delivery of high-dose nisin gel had no adverse effect on the expression of TLRs-3 and 9 compared to the placebo gel-treated group.

### 3.2. Flow cytometry of TLRs

**Figure 2:**
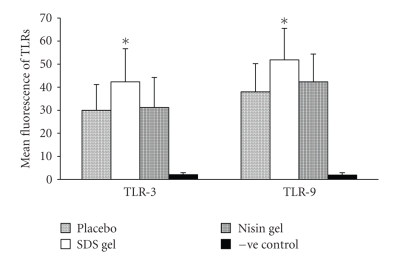
Flow cytometric analysis of TLR expression in rabbit CVE cells after intravaginal administration of placebo, nisin (15.15 mM in 2 mL of 1% polycarbophil gel) SDS (56 *μ*M in 2 mL of 1% polycarbophil gel) and 2 mL of 1% polycarbophil gel. Mean fluorescence intensity for TLR-3 and 9 were significantly increased in SDS gel-treated animals compared to their respective placebo and nisin gel-treated groups. The mean and error bars indicate standard deviation of triplicate measurements obtained from three separate experiments.

The inflammatory potential of nisin gel was further evaluated by flow cytometry. The results indicated no significant difference in the number of cells positive for TLR-3 and 9 between placebo and nisin gel-treated groups (Figure 2). However, in the SDS gel-treated group, mean fluorescence intensity for TLR-3 and 9 increased significantly. The experiment was repeated twice in duplicates and similar results were obtained.

### 3.3. RT-PCR analysis of TLR expression

**Figure 3:**
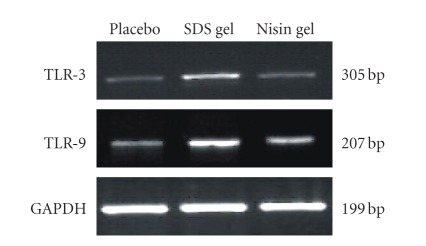
RT-PCR analysis of TLR- 3 and 9 expression. Total RNA was extracted from CVE cells and subjected to RT-PCR analysis as described in the Material and Methods. No change in TLR gene expression was observed in nisin administered animals compared to placebo gel-treated group. However, in SDS gel administered animals, the expression of TLR genes was upregulated. House keeping gene, GAPDH expression confirms roughly equivalent loading of RNA samples.

To determine whether vaginal administration of nisin gel leads to modulation of TLRs and cytokine genes, expression of TLR and cytokine mRNA transcripts were analyzed using RT-PCR. As shown in Figures 3 and 9, RT-PCR results demonstrated no change in the expression in nisin gel-administered rabbits compared to the placebo group.

### 3.4. Immunohistochemical localization of cytokines

**Figure 4:**
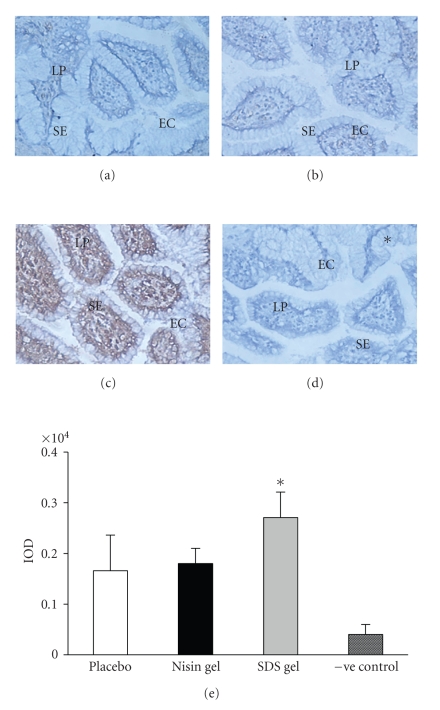
Immunohistochemical localization of IL-4 in the CVE after intravaginal application of nisin (15.15 mM in 2 mL of 1% polycarbophil gel) (b), SDS (56 *μ*M in 2 mL of 1% polycarbophil gel) (c) and 2 mL of 1% polycarbophil gel (a) for 14 consecutive days. To rule out the nonspecific binding, sections were incubated in preimmune sera instead of primary antibody and considered as negative control (d). Semi-quantitative comparison by measuring integrated optical density (IOD) of immunoreactive IL-4 in treated and placebo is shown (e). The IOD in each area was calculated for the brown color immunoprecipitates. Each IOD value measured is the mean ± SD of six observations obtained from three independent samples. (Mag X 20) (EC = epithelial cells, SE = Squamous epithelium, LP = lamina propria), (* = value is statistically significant at *P* < .05).

**Figure 5:**
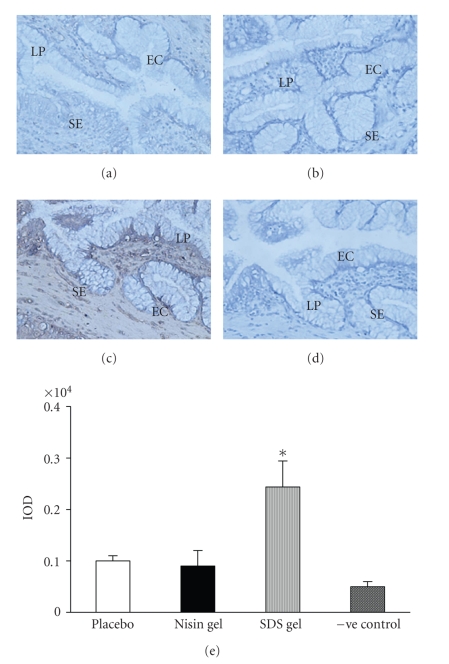
Immunohistochemical localization of IL-6 in the cervicovaginal tissue after intravaginal application of nisin (15.15 mM in 2 mL of 1% polycarbophil gel) (b), SDS (56 *μ*M in 2 mL of 1% polycarbophil gel) (c) and 2 mL 1% polycarbophil gel (a) for 14 consecutive days. To rule out the nonspecific binding, sections were incubated in preimmune sera instead of primary antibody and considered as negative control (d). Semiquantitative comparison by measuring integrated optical density (IOD) of immunoreactive IL-6 is shown (e). The IOD in each area was then measured for the brown color immunoprecipitates. Each value is the mean ± SD of six observations obtained from three independent samples. The figure shown is representative pictures from three independent rabbits (Mag X 20). (EC = epithelial cells, SE = squamous epithelium, LP = lamina propria) (* = value are statistically significant at *P* < .05).

**Figure 6:**
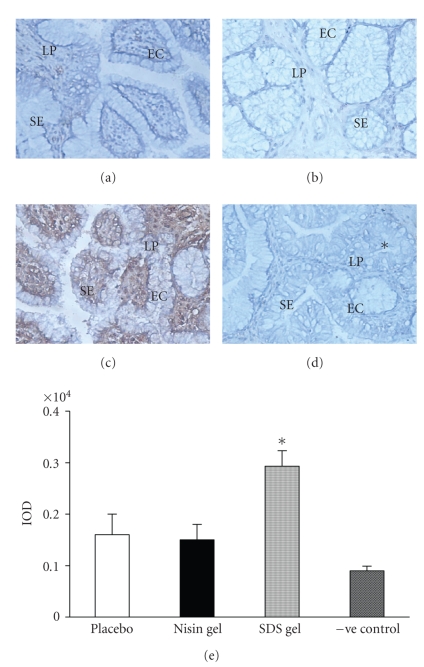
Immunohistochemical localization of IL-10 in the CVE after intravaginal application of nisin gel (15.15 mM in 2 mL of 1% polycarbophil gel) (b), SDS (56 *μ*M in 2 mL of 1% polycarbophil gel) (c) and 2 mL of 1% polycarbophil gel for 14 consecutive days. To rule out the nonspecific binding, sections were incubated in preimmune sera instead of primary antibody and considered as negative control (d). Semiquantitative comparison by measuring integrated optical density (IOD) of immunoreactive IL-10 in treated and placebo is shown (e). The IOD in each area was then measured for the brown color immunoprecipitates. Each value is the mean ± SD of six observations obtained from three independent samples. Figure shown is representative picture from three independent experiments. (Mag X 20) (EC = epithelial cells, SE = squamous epithelium, LP = lamina propria), (* = values are statistically significant at *P* < .05).

**Figure 7:**
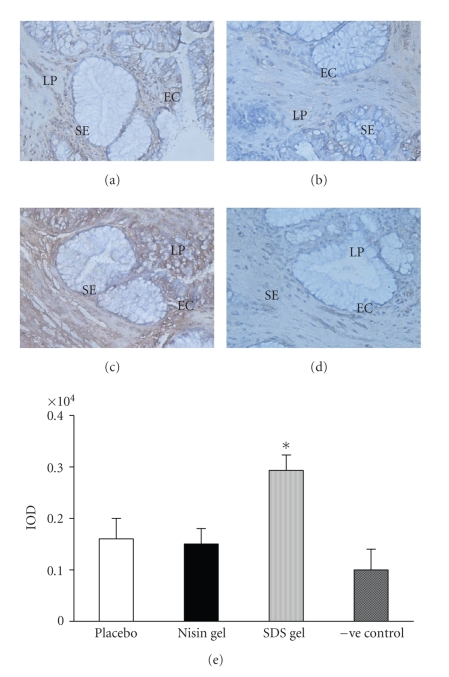
Immunohistochemical localization of TNF-*α* in the cervicovaginal tissue after intravaginal application of nisin (15.15 mM in 2 mL of 1% polycarbophil gel) (b), SDS (56 *μ*M in 2 mL of 1% polycarbophil gel) (c) and 2 mL of 1% polycarbophil gel (a) for 14 consecutive days. To rule out the nonspecific binding, sections were incubated in preimmune sera instead of primary antibody and considered as negative control (d). Semiquantitative comparison by measuring integrated optical density (IOD) of immunoreactive TNF-*α* in treated and placebo is shown (e). The IOD in each area was then measured for the brown color immunoprecipitates. Each value is the mean ± SD of six observations obtained from three independent samples. Figure shown is representative picture from three independent experiments. (Mag X 20) (EC = epithelial cells, SE = squamous epithelium, LP = lamina propria) (* = values are statistically significant at *P* < 0.05).

**Figure 8:**
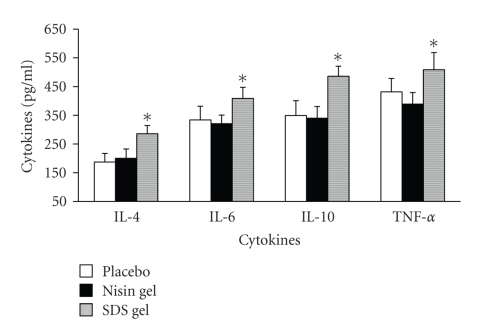
Levels of cytokines (IL-4, IL-6, IL-10 and TNF-*α*) in CVL after intravaginal application of nisin (15.15 mM in 2 mL of 1% polycarbophil gel) SDS (56 *μ*M in 2 mL of 1% polycarbophil gel) and 2 mL of 1% polycarbophil gel for 14 consecutive days. CVL are collected on day 15, separated from cell debris by centrifugation and used for cytokine determination as per the protocol given in the material and methods section. Each value is the mean ± SD of six observations from three separate experiments. No significant difference in cytokine levels was observed between placebo and nisin gel treated groups. In contrast the levels were significantly increased in SDS gel treated animals (* = values are statistically significant at *P* < 0.05).

**Figure 9:**
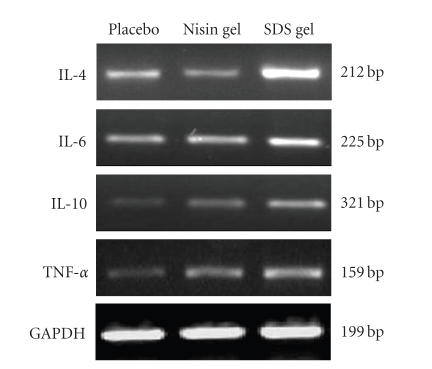
RT-PCR analysis of cytokine expression following intravaginal application of nisin (15.15 mM/day/14 days), SDS (56 *μ*M/day/14 days) and 1% placebo gels in rabbits. After separation of cells, they were subjected to RNA extraction as per the protocol given in the material and methods section. The expression of IL-4, IL-6, IL-10 and TNF-*α*. genes in nisin treated group was comparable to the placebo group. In contrast, expression of the cytokine genes was upregulated in SDS treated group. GAPDH blots confirmed roughly equivalent loading of RNA samples.

Having demonstrated that CVE cells express TLRs-3 and 9, we next investigated the modulation of various cytokines in cervicovaginal tissue in response to microbicide gels by immunohistochemistry. The expression of cytokines (IL-4, 6, 10, and TNF-*α*) was observed in the cytoplasm as secreted products with no change in staining intensity between placebo and nisin-treated groups. However, the levels were significantly upregulated (Figures 4–7) in SDS treated animals. This increase was not associated with enhancement in the number of leucocytes or neutrophils in CVLs (data not shown). ELISA results of CVLs were in perfect agreement with immunohistochemical data (Figure 8).

### 3.5. Determination of NF-*k*B P65 in CVE cells

**Figure 10:**
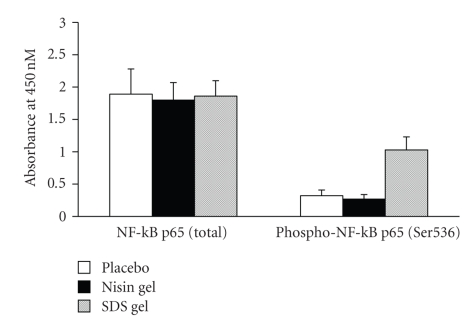
Determination of total and phosphorylated NF-*k*B p65 protein levels by ELISA as per the protocol described in materials and methods. No change was observed in total NF-*k*B levels. However, the phosphorylated NF-*k*B protein levels were significantly elevated in SDS gel administered animals.

Modulation of NF-*k*B-related signals was determined after intravaginal application of nisin gel and compared with placebo gel-administered group. The phosphorylation state of NF-*k*B and consequent changes in gene expression following NF-*k*B modulation were determined by ELISA by comparing relative amounts of phosphorylated NF-*k*B protein to the total NF-*k*B protein. Exposure of CVE to SDS gel triggered a marked activation of NF-*k*B. However, such induction in NF-*k*B signalling was not observed in nisin gel treated animals and the placebo gel-treated group (Figure 10).

### 3.6. RT-PCR analysis of rabbit defensin-1

**Figure 11:**
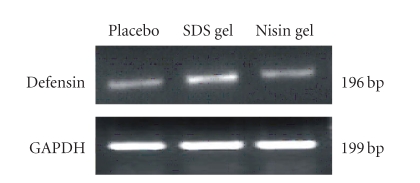
RT-PCR analysis of defensin-1 gene in CVE cells of rabbit. No change was seen in defensin-1 gene expression between nisin gel and placebo gel administered groups. However, significant activation of defensin-1 gene expression was observed in SDS gel administered group compared to placebo and nisin gel treated animals.

To examine whether the observed increase in cytokine levels is associated with changes in vaginal mucosal immunity, we evaluated the expression of defensin-1 gene in CVE cells. The results from three independent experiments revealed that nisin gel treatment did not cause any alterations in the mRNA expression of defensin-1 compared to placebo gel-treated group. Whereas in SDS gel treated group expression of defensin-1 gene was markedly elevated (Figure 11).

## 4. DISCUSSION

Topical microbicides are being evaluated as promising strategies for the prevention of STIs/HIV while protecting or enhancing the natural mucosal barrier [27, 28]. Nisin, a naturally occurring AMP, is currently being developed as an intravaginal microbicide for the prevention of STIs and unintended pregnancies [8]. To screen nisin gel for undesirable characteristics if any in a preclinical setting, we chose the rabbit model, since it has been documented that responsiveness of rabbit vagina to microbicides resembles that of the human vagina [29].

Immunomodulation inflammation has been implicated to play a role in vaginal innate immunity [30]. Inflammatory responses limit the beneficial effect of microbicides, which could be of particular concern if the inflammation persists beyond the time the microbicide was present [29]. Vaginal epithelial cells represent the predominant resident innate immune cell in the vagina and may modulate the inflammatory response to microbicides [31]. Mammalian Toll-like receptors (TLRs), including TLR-1 to TLR11, serve an important role as sensors in host defense mechanism [32]. On the basis of TLR role in the modulation of innate immunity and responses to inflammatory challenge, we examined the expression of TLR-3 and 9 as these play a crucial role in the recognition of inflammatory stimuli in host vaginal immune defense [33, 34].

Under conditions of their intended use as intravaginal/rectal microbicides, individuals would be exposed to microbicides for shorter or longer periods. Therefore, high dose of nisin gel (15.15 mM in 2 mL of 1% polycarbophil) per day for 14 consecutive days was administered in rabbits to ensure whether the estimated risk/benefit profile of the gel is in favor of its intended use. The immunofluorescent results demonstrated localization of TLRs-3 and 9 on the plasma membrane as well as in endosomes present in the cytoplasm of CVE cells. TLR expression did not change in the CVE cells of nisin and placebo gel-treated groups. These results were in agreement with the flow cytometry data where no change in percent immunoreactive cells expressing TLRs-3 and 9 was observed. However, the number of cells expressing both TLRs-3 and 9 were significantly elevated in SDS gel-treated animals. The studies were extended further to gain an insight into the regulation of TLR expression at the molecular level. RT-PCR results demonstrated no change in the expression of TLRs-3 and 9 mRNA transcripts in CVE cells of nisin gel-treated rabbits compared to the placebo gel-treated group, suggesting that vaginal application of nisin gel did not induce any inflammation associated alterations at the transcriptional and translational level. The CVE cells recognize tissue inflammation by means of TLR-3 and 9, likely through a nonspecific mechanism. These results tempted us to speculate that signalling through TLRs may be involved in mechanisms provoking Th2-based immune responses and may either prevent or promote vaginal inflammation [35].

Earlier studies have suggested that many of the local inflammatory responses initiated in the vaginal mucosa are mediated by a complex interplay between TLRs and inflammatory agents in the human vagina [32, 36]. In an elegant study, Fichorova et al. [33] have shown the presence of TLR 1-6 genes in primary and immortalized endocervical, ectocervical, and vaginal epithelial cell lines. They demonstrated mRNA for TLRs-1, 2, 3, and 6 in these cells. In contrast, no PCR product was detected for TLR-4 in either of the cells. It has been shown recently that agonists of TLR-2 (zymogen), TLR-3 [poly (I: C)], TLR-5 (flagellin), and TLR-9 (CpG ODN) are known to stimulate various immune cells to secrete several proinfammatory cytokines [23]. These studies support our present observations that nisin gel did not upregulate the levels of inflammatory cytokines secreted by CVE cells. In contrast, in SDS gel-treated animals, the levels of cytokines in the vaginal tissue and vaginal fluid were upregulated in a TLR-3/9-dependent manner probably to slow down the tissue inflammation.

To further dissect the mechanism, we next investigated the expression of NF-*k*B, known for its ability to regulate cytokine and defensin-1 expression in CVE cells. As shown in Figure 10, the phospho NF-*k*B p65 and total NF-*k*B p65 remained unchanged in placebo and nisin gel-treated groups, suggesting that vaginal application of nisin gel did not alter the NF-*k*B signalling cascade.

We further evaluated the expression of defensin-1 which is the principal effector molecule of mucosal innate immunity against infection/inflammation [37]. Considering the fact that several defensins are expressed by neutrophils, epithelial cells, macrophages, NK cells, and T cell subsets in human [38, 39], the present results indicated that, nisin gel treatment did not cause any change in the expression of defensin-1 gene suggesting that the NF-*k*B signalling cascade was not affected. The aforementioned studies demonstrated that nisin gel may either enhance or maintain the normal immune status to assist in the resolution of inflammation, while SDS gel appears to cause immunosuppressive effect.

Therefore, we hypothesized that TLRs or cytokines appear to be predictive biomarkers for evaluating the inflammatory status of vaginal mucosa upon microbicide administration. These results are supported by the fact that soluble TLRs-2 and 4 modulate specific TLR-mediated responses, thereby providing new biomarkers of tissue inflammation [40]. The present data led us to postulate that TLRs appear to be crucial mediators of vaginal defense and when perturbed cause inflammatory pathways to predominate in CVE cells leading to mucosal damage. These findings contribute to our understanding of mucosal immunity against STIs/HIV and can aid in enhancing the quality of woman's reproductive health. Establishing downstream targets for TLR–microbicide interaction may further unravel the intricacies of vaginal function.

The present study is the first to provide evidence of nisin gel as a safe intravaginal compound for future use in humans. Testing additional compounds in the presently planned clinical trials would allow validation of this approach and determine whether this safe and inexpensive strategy provides a surrogate biomarker to predict microbicide safety and efficacy. However, this study is too small to make a definite conclusion. If confirmed in a larger sample, this strategy could be used to determine which future generation microbicides should go forward in development, possibly resulting in major cost savings. However, given the nascent state of knowledge concerning this important area, it is clear that more studies are needed to provide valuable insights into the biology of TLRs and vaginal innate immunity.

## Figures and Tables

**Table 1:** Oligonucleotide primers used for the study.

Gene	Accession No	Primer sequence (5′→3′)	Product size (bp)	Annealing temperature
IL-4	DQ_852343	F-AGTTCTACCTCCACCACAAGGT	212	60°C
		R-TCAGCTCTGACGCTTTGAGTAT		

IL-6	NM_001082064	F-AGGAGCTGAGGAAAGAGATGTG	225	58°C
		R-TGTTTTCTTCGTCACTCCTGAA		

IL-10	NM_001082045	F-TTCTTTCAATCGAAGGATCAGC	267	59°C
		R-CTCATTCATGGCTTTGTAGACG		

TNF-*α*	ID_AB128153	F-CTAGCCCACGTAGTAGCAAACC	159	60°C
		R-GCTGAAGAGAACCTGGGAGTAG		

TLR-3	NM_003265	F-GAT CTG TCT CAT AAT GGC TTG	305	60°C
		R-GAC AGA TTC CGA ATG CTT GTG		

TLR-9	NM_017442	F-TTC CCT GTA GCT GCT GTC C	207	59°C
		R-ACA GCC AGT TGC AGT TCA CC		

Defensin-1	NM_001082299	F-CTCTGCTTGCTGCCATTCTC	196	60°C
		R-AATCGTCTGCAAGTACAGGACAC		

GAPDH	NM_01082253	F-CCATTCATTGACCTCCACTACA	199	59°C
		R-CGTTGCTGACAATCTTGAGAGA		
